# Do airway metallic stents for benign lesions confer too costly a benefit?

**DOI:** 10.1186/1471-2466-8-7

**Published:** 2008-04-18

**Authors:** Andrew L Chan, Maya M Juarez, Roblee P Allen, Timothy E Albertson

**Affiliations:** 1 University of California, Davis Medical School, Department of Internal Medicine, Division of Pulmonary and Critical Care Medicine, Sacramento, USA

## Abstract

**Background:**

The use of self-expanding metallic stents (SEMAS) in the treatment benign airway obstruction is controversial.

**Methods:**

To evaluate the safety and efficacy of SEMAS for this indication, we conducted a 10-year retrospective review at our tertiary medical centre.

**Results:**

Using flexible bronchoscopy, 82 SEMAS (67% Ultraflex, 33% Wallstent) were placed in 35 patients with inoperable lesions, many with significant medical comorbidities (88%). 68% of stents were tracheal, and 83% of patients showed immediate symptomatic improvement. Reversible complications developed in 9% of patients within 24 hrs of stent placement. Late complications (>24 hrs) occurred in 77% of patients, of which 37% were clinically significant or required an interventional procedure. These were mainly due to stent migration (12.2%), fracture (19.5%), or obstructive granulomas (24.4%). The overall granuloma rate of 57% was higher at tracheal sites (59%) than bronchial ones (34%), but not significantly different between Ultraflex and Wallstents. Nevertheless, Wallstents were associated with higher rates of bleeding (5% vs. 30%, p = 0.005) and migration (7% vs. 26%, p = 0.026). Of 10 SEMAS removed using flexible bronchoscopy, only one was associated with incomplete removal of fractured stent wire. Median survival was 3.6 ± 2.7 years.

**Conclusion:**

Ill patients with inoperable lesions may be considered for treatment with SEMAS.

## Background

The role of stent placement for the treatment of benign airway obstruction is still being fully elucidated. On July 29, 2005, the US Food and Drug Administration (FDA) issued a Public Health Notification recommending against the use of both covered and uncovered self-expanding metallic tracheal stents (SEMAS) for benign lesions unless absolutely necessary [1]. Lund et al's recent editorial also counselled restraint when considering the placement of SEMAS for benign lesions [2]. These concerns were based on reports of obstructing granulomas, mucous plugging, stent fracture, migration, and infection. Multiple authors have reported such complications [3-6]. In contrast, Thornton and colleagues [7] reported that with SEMAS, assisted patency rates of nearly 90% could be achieved, and that SEMAS were well tolerated for years, and beneficial in patients with selected benign indications. Husain et al [8] found Ultraflex stents to be both safe and effective in the prolonged palliation of benign airway obstruction.

Concern regarding the potential for stent removal to cause mucosal tears, haemorrhage, and even pneumothoraces was also raised. However, Noppen et al [9] found that covered stents could be removed effectively and safely without major complications.

In view of the differing opinions in the published literature, we retrospectively reviewed the long-term effects of SEMAS placed in the airways for benign indications at our university medical centre over a 10 year period. In particular, we applied stringent criteria to detect complications related to metallic stent placement, removal and their sequaelae.

## Methods

Data were retrospectively obtained from medical records of patients who underwent SEMAS placement for the treatment of non-malignant indications at our institution, a 577-bed tertiary-care teaching hospital. Patients who were treated from January 1996 to March 2006 were included in this review. Patients with malignant airway obstruction were excluded.

The data collected included patient demographics; indications for SEMAS placement; comorbidities at presentation; recorded symptoms of airway obstruction immediately prior to, within 24 hours of, and between 24 hrs and 12 weeks. Symptoms of airway obstruction were classified as respiratory distress (including shortness of breath, progressive dyspnea, respiratory distress, and hypoxemia), cough, stridor, difficulty clearing secretions, and obstructive pneumonia. Also noted were elective or emergent indications for SEMAS placement, bronchoscopic mode (flexible or FB vs. rigid or RB), bronchoscopic venue (Intensive Care Unit or ICU, bronchoscopy suite, or operating room), stent type (covered vs. uncovered), stent brand (Microvasive Ultraflex vs. Wallstent Endoprosthesis, both by Boston Scientific; Natick, MA) and size (length and diameter). Complications of SEMAS treatment and patient mortality were also recorded. Patient mortality was assessed through a search of our medical records, the Social Security Death Index, and via a search of death records by the California Department of Health Services.

One of two brands of SEMAS was chosen depending on physician preference and availability. A covered stent was chosen if the potential for continued granulation tissue was considered significant. All stents were placed by four pulmonary and critical care specialists. Patients underwent flexible or rigid bronchoscopy using conscious sedation or general anesthesia respectively, depending on the individual patient's clinical status as assessed by the interventionalist of record. General anesthesia was administered during RB utilizing propofol. Conscious sedation was achieved using fentanyl and midazolam titrated to effect during FB, in addition to topical lidocaine anesthesia.

The technique for SEMAS removal depended on the extent of epithelization of the target stent. In the main, an attempt was made to remove the stent in its entirety if deemed safe. If, in the opinion of the operator, this was not feasible, the stent was unraveled or removed piecemeal, sometimes utilizing an Nd:YAG laser.

Statistical analyses were performed using JMP software (SAS Institute; Cary, NC). The proportion of complications associated with stent location, types, and brands were compared using χ^2 ^or Fisher's exact test. Logistic regression was employed to determine the significance of the influence of stent brand and type on complication rate. Survival was assessed using Kaplan-Meier analysis. Death from any cause was considered an event, and data on patients who were alive at the study conclusion were considered censored. A p-value < 0.05 was considered significant. Mean and median statistics are reported ± standard deviation.

This study was conducted with the approval of our Institutional Review Board and in compliance with the Health Insurance Portability and Accountability Act.

## Results

During a 10-year period, 35 patients underwent 62 bronchoscopies resulting in the placement of 82 SEMAS. The mean number of SEMAS placed per patient was 2.3 ± 1.5, with a range of 1 to 7. Seventeen patients were female. Mean patient age was 59.5 ± 14.3 years (range = 28 to 83 years).

All patients were either deemed medically unsuitable for surgery by the interventionalist or had declined a surgical solution. Thirty-four (97%) patients had multiple indications for stent placement (Table 1). The indications most frequently occurring together were tracheobronchomalacia and postintubation stenosis (n = 9 patients). Medical comorbidities were commonly seen (89% of patients, Table 2). Presenting symptoms were frequently multiple (76%) and included symptoms of airway obstruction (Table 3). Most patients (89%, n = 31) underwent at least one intervention to treat airway obstruction prior to SEMAS placement (Table 4).

**Table 1:** Indications for SEMAS placement

	n (patients)	% total patients
Tracheobronchomalacia	23	65.7
Postintubation stenosis	17	48.6
Lung transplant anastomosis stenosis	4	11.4
Tuberculosis or mucormycosis scarring	3	8.6
Radiotherapy changes	2	5.7
Localized cartilaginous hypertrophy	1	2.9
Wegner's granulomatosis	1	2.9
Bronchial necrosis due to foreign matter aspiration	1	2.9

**Table 2:** Comorbidities at presentation.*

	n (patients)	% total patients
Chronic obstructive pulmonary disease	14	40.0
Pneumonia	10	28.6
Lung transplant	4	11.4
Interstitial lung disease	4	11.4
Cerebro-vascular accident	3	8.6
Adult respiratory distress syndrome	2	5.7
Other infection (non-respiratory)	2	5.7
Congestive heart failure	2	5.7
Other malignancy	1	2.9
Collage-Vascular Disease	1	2.9
Immunocompromised	1	2.9
Critical illness neuromyopathy	1	2.9

*Patients may have had more than 1 comorbidity. Comorbidities were observed in 89% (n = 31) of patients.

**Table 3:** Symptoms of airway obstruction at presentation.

	n (patients)	% total patients
Respiratory distress	29	82.9
Cough	17	48.6
Stridor	14	40.0
Difficulty clearing secretions	13	37.1
Obstruction pneumonia	1	2.9

**Table 4:** Therapeutic interventions prior to SEMAS placement

	n (patients undergoing intervention)	% of 31 patients undergoing at least 1 intervention
Laser resection (Nd:YAG, KTP, or CO2)	24	77.4
Balloon dilatation	16	51.6
Silicone stent placement	5	16.1
Tracheostomy tube placement	4	12.9
Bronchoscopic debridement	2	6.5

The majority of SEMAS placement bronchoscopies were performed in a bronchoscopy suite (71%, n = 44) or ICU (23%, n = 14), and were elective or non-emergent (92%, n = 57). Of 5 emergent bronchoscopies, 4 were performed in the ICU. Another 4 bronchoscopies (6%) were performed in the operating room, utilizing RB in three. Of 59 FB's, 53 (90%) were performed using conscious sedation.

Fifty-five of 82 (67%) SEMAS were Ultraflex, and 69% of these were uncovered. Of 27 Wallstents used, 59% (n = 16) were uncovered. Mean SEMAS length was 40 ± 10.6 mm (range = 20–60 mm), and mean diameter was 16 ± 3.3 mm (range = 8–22 mm).

SEMAS were most frequently deployed in the trachea (68% of stents, n = 56), and were mainly proximal or subglottic (40%, n = 33) (Table 5). Eighty percent of patients (n = 28) underwent additional interventions *post *airway stent placement to treat SEMAS-related complications or as part of a surveillance protocol (Table 6).

**Table 5:** Stent deployment location.

	n (stents)	% total stents
Proximal trachea/subglottic	33	40.2
Mid trachea	11	13.4
Distal trachea	12	14.6
Left main bronchus	17	20.7
Right main bronchus	6	7.3
Right broncus intermedius	3	3.7

Proximal trachea = upper third, mid-trachea = middle third, distal trachea = lower third.

**Table 6:** Additional interventions post SEMAS placement:

	N (patients)	% of 28 patients undergoing at least one intervention post metallic stent placement	Range (# procedures per patient)
Repeat bronchoscopy (unrelated to procedures below)	27	96.4	0–13
Laser resection	12	42.9	0–4
Balloon dilation	4	14.3	0–4
Tracheostomy tube placement	1	3.6	0–1
Debridement of granulation tissue	1	3.6	0–1
Silicone stent placement after failure of metallic stent	1	3.6	0–1

Symptomatic improvement within 24 hours of stent placement was noted in 83% of patients. In particular, stridor (73% of cases); respiratory distress (71%); cough (79%); and difficulty clearing secretions (75%) were noted to have resolved or improved.

The mean time to first repeat bronchoscopy post SEMAS placement was 173 ± 267 days (median = 37 days), with a mean number of additional therapeutic bronchoscopies of 4.3 ± 2.9 (median = 4 interventions).

Overall, 80% of patients (n = 28) experienced one or more complications. A total of 172 complications were observed. Complications were categorized as early (occurring within 24 hours post stent placement) and late (occurring > 24 hours post stent placement), and also as major and minor. Major complications were defined as those requiring an intervention or having a significant clinical impact. Minor complications were defined as those not necessarily requiring an intervention or having significant clinical impact. Forty-six percent of patients (n = 16) experienced at least one major complication and 77% (n = 27) experienced one or more minor complications.

### Early complications

Three patients (9%) experienced major complications: one patient who had received laser phototherapy immediately prior to stent placement developed stridor from glottic edema requiring temporary intubation for resolution several hours after the procedure; another experienced sustained desaturation to 83% and persistent cough which resolved after 3 hours of continuous positive airway pressure (CPAP) therapy and nebulized albuterol treatments. In the third patient, a single stent migrated distally requiring removal and replacement with a silicone stent (Dumon, Novatech, La Ciotat, France).

### Late complications

Late complications occurred in over three-fourths (77%) of patients; with major complications occurring in 37% (n = 13) of patients, and minor complications in 77% of patients (n = 27) (Table 7). Overall, granulation tissue formation (obstructive or non-obstructive) at the site of the SEMAS was observed in 57% (n = 20) of patients, and was significantly higher in tracheal (59%, n = 33 of 56) than in bronchial (34%, n = 9 of 26) stents (p = 0.040).

**Table 7:** Complications occurring >24 hrs post stent placement*:

	n (stents associated with complication)	% total stents	n (patients)	% total pts
Major complications				
Obstructive granuloma	20	24.4	10	28.6
Stent fracture	16	19.5	6	17.1
Stent migration	10	12.2	6	17.1
Minor complications				
Impaired secretion clearance	34	41.5	17	48.6
Granulation tissue formation overlying stent	13	15.8	13	37.1
Infection (local or pneumonia)	17	20.7	8	22.9
Bleeding (not requiring transfusion)	11	13.4	6	17.1
Persistent cough	10	12.2	6	17.1
Stent compression or misshaping	4	11.5	4	11.4
Disease recurrence at stent site	6	7.3	3	8.6

*More than one complication may have occurred per patient.

Progressive dyspnoea occurred in 11 (31.4%) patients >24 hrs post stent placement; but were assessed to have been related to the patients' comorbidities. Neither airway perforation nor fistula formation complicated SEMAS placement.

No significant difference in complications or survival was observed between stent types. However, Wallstents were associated with a significantly higher proportion of late complications compared to Ultraflex SEMAS, in particular, bleeding (30% vs. 5%, P = 0.005) and migration (26% vs. 7%, P = 0.026).

Median length of patient follow-up post SEMAS placement was 473 ± 654 days (mean = 623.5 days). The majority of in-situ stents (88%) as of last known patient follow-up, had remained in the airways for a mean of 616 ± 654 days (range = 1 to 3205 days).

Removal of 12 SEMAS was attempted in 4 patients; 10 were successfully removed. Indications for removal included stent fracture (n = 6), migration (n = 5), granulation tissue (n = 4), mucous plugging/obstruction (n = 4), recurrence of stenosis (n = 3), no clinical improvement (n = 3), and hemoptysis (n = 1). Both stents that could not be removed were uncovered; one was a Wallstent, the other an Ultraflex stent. In one patient, a stent was too deeply embedded in the endotracheal mucosa for safe removal. In another, a single stent could not be removed despite manipulation. This patient subsequently expired from causes unrelated to stent placement. Nine of 10 (90%) stent removals were uncomplicated. The single complication observed was the retention of a fractured stent wire that was too deeply embedded in the mucosa.

Half of the 12 SEMAS selected for removal were covered. Four were Wallstents, and 8 were Ultraflex. The longest indwelling stent removed had been in place for 3.8 years.

Twenty patients (57%) were alive up to the date of this review (April 2007). Median survival post initial SEMAS placement was 3.6 ± 2.7 years, range 10 days to 10.3 years.

While no deaths could be directly attributed to the airway stents, the exact cause of death for 8 subjects in this review could not be determined despite strong data collection efforts. Thus, it is possible that the death rate in this series may be as high as 23% (8 of 35 patients). The median survival time of patients for whom cause of death could not be determined was 2.4 ± 1.2 years.

## Discussion

Patients with benign stenotic airways disease should be considered for surgical intervention [3]. We have, however, focused in this 10 year retrospective study on a cohort of 35 patients with benign airway lesions who were not surgical candidates because of significant medical comorbidities (88%), or because they declined surgery. While the majority of our stents (n = 77, 91%) were placed prior to the FDA's Public Health Notification [1], we suggest that similar groups of highly selected patients may benefit from SEMAS placement [8] Indeed, most (90%) of our patients had undergone an interventional bronchoscopic procedure prior to stent placement, mainly laser phototherapy (77%), and/or balloon dilatation (52%), consistent with the acuity and severity of their disease. Ninety-seven percent were symptomatic, 4 required prior temporizing tracheostomy tube placement, and another five required SEMAS to replace silicone stents (Dumon) that had mostly migrated.

SEMAS placement was highly effective, affording symptomatic improvement in 83% of patients within 24 hours. In particular respiratory distress/stridor and cough were alleviated after a mean of 2.3 stents were placed in each patient. These results are supported by other authors [7,10].

While the majority (94%) of our stents were placed using FB and conscious sedation, only 5% were placed using RB because of the patients' clinical status, operator preference, and logistic issues. Utilisation of the FB for metallic stent placement appears to be a safe method under these circumstances [11], with 68% of stents in our series being placed in the trachea, especially proximally, and, 21% in the left main bronchus.

The use of silicone stents in benign disease does not appear to be as controversial as that of SEMAS [12]. Nevertheless, we chose SEMAS over silicone stents because of a superior inner diameter to wall thickness ratio [8,13], and potentially fewer problems with stent migration [10,14,15] and airway obstruction due to secretions[16]. Some of our patients were deemed medically unsuitable for general anaesthesia and hence RB by the interventionalist of record. Silicone stents are easily removed after placement, even several years later [17], but as Walser [18] suggests, they have a potential for migration in up to 28% of cases. In addition, Grewe et al [19] found that airway non-covered metal stents may be utilized long-term for nonmalignant obstructive disease, and that no malignant transformation of previously non-tumorous tissue occurred.

No patient in our series was complicated by airway perforation or tracheo-oesophageal fistulae resulting from SEMAS placement. Initial technical success in reference to metallic stent placement was 100%. However, during the first 24 hours post initial stent placement, three patients (9 %) experienced major reversible complications that were ultimately resolved by additional interventions. The immediate complication rate appears similar to that of Thornton et al [7].

Late complications occurred in 77% of our patients, with this figure reflecting relatively minor complications in 27 patients. However, 13 patients, (37%) experienced major complications, mainly because of stent migration and/or fracture, or for treatment of obstructive granulomas. In comparison, a total of 28 patients (80%) required an interventional procedure after SEMAS placement that was not necessarily related to complications from SEMAS. This higher proportion of patients likely reflects the course of the patients' disease process, especially in terms of impaired mucous clearance. The time to first repeat bronchoscopy was highly variable, again likely reflecting the individual patient's disease course, with a mean of 6 months. A majority (88%) of SEMAS remained in-situ as of last known patient follow-up, representing a mean of 23.8 ± 21.8 months.

Although Saad et al [16] found no difference in complication rates between Wallstent and Ultraflex stents, or covered and uncovered ones; our series found that Wallstents were associated with a significantly higher incidence of bleeding and migration. While the explanation for this is unclear, and may be related to the individual patient's anatomy and disease process, the somewhat different structures of both stents are noted. These complications, however, were only observed in 13% of stents. Rieger et al [20] found that Wallstents led to the formation of granulomas that required frequent intervention. While granulation tissue did occur in all our stent types, no significant difference was found in its incidence amongst the stent categories. Our finding of granulation tissue in 57% of patients is greater than that reported by Saad et al for benign conditions (33.3%) [16]. Apart from individual patient differences especially in terms of bronchial mucosal inflammation, our longer median follow up of 473 days vs. 336 days may have also contributed to the higher figure. We did note however that 40% of our stents had been placed in the proximal/subglottic trachea, where 16 of 20 (80%) patients developed granulomas. Based on this result, we agree that the placement of stents in the proximal/subglottic trachea should be avoided if at all possible.

While we have included Wallstents in this retrospective, it is important to note that the manufacturer of this type of stent does not now recommend their use in the airways.

Several authors have suggested that removal of metallic airway stents are ideally performed using RB and general anaesthesia [9,21], or using a combined open and endoscopic approach [22]. Our data suggest that suitably selected patients can safely undergo SEMAS removal utilizing a FB. This appears similar to Thornton et al's [7] findings where the authors did not use RB for successful stent removal. Of the two SEMAS that could not be removed in our study, only one was deemed too risky to be removed. Nevertheless, we agree with Noppen et al [9] that under certain circumstances the removal of SEMAS that has established in the airway mucosa can be very hazardous, requiring much forethought prior to removal.

Our study is limited by its retrospective nature and is best viewed as a single tertiary referral centre's experience in managing a cohort of highly symptomatic and ill patients. Surgical reconstruction, especially of the trachea, can be technically challenging, and may of itself lead to anastomotic dehiscence and re-stenoses [23]. Kabbani et al [23] suggest that stent placement can be an attractive alternative even in benign airway obstruction for these reasons. The exact indications for stent placement in benign strictures are still being emotionally debated amongst pulmonologists and surgeons [24], appear institution specific, but at least include airway compression from benign thyroid disease [25] and strictures from transplantation anastomoses [26].

Of interest, all mortalities in this series occurred within 3.5 years of stent placement. This finding may reflect the significant co-existing medical morbidities in our patient cohort. Although we have attributed the deaths of 8 of those patients to complications resulting from SEMAS placement, this was done by convention because of lack of available data as to their actual cause of death.

Comparison to other series is difficult not least because of differing patient pathologies and their severities [7,16,27]. Madden et al [3] reported that 48% of their patients had at least one if not more large airway complications from Ultraflex stent placement, with granulation tissue occurring in 35% of patients. In our series, 80% of stents, representing 80% of patients, were linked to at least one complication. While very rigorous and inclusive in our data collection, we found that major complications, although associated with 37% (n = 30) of stents in 46% of patients, were largely reversible. Nevertheless, we agree with other authors [2,3,7,16,20,28] that other surgical and medical therapeutic options should be first considered, including perhaps non-invasive positive pressure ventilation. Stent placement was the last resort in our patient cohort to effectively relieve symptoms. Certainly, silicone stents are currently reported to be preferable to SEMAS for benign lesions, and need to be strongly considered. However, neither silicone stents nor SEMAS are ideal, and some patients would not have been able to tolerate general anaesthesia for RB. Emad [29] suggests that the ideal stent in the future would be made of a biocompatible material that maintains mucociliary clearance and minimizes granulation tissue formation. Saito et al [30] report on a new tubular bioabsorbable stent made of poly-L-lactic acid in the rabbit model, and Zakaluzny et al [31] discuss drug-eluting stents to inhibit granulation tissue. Together with improved techniques in the stent insertion and removal, and possibly surveillance bronchoscopies, morbidity and mortality may be minimized [16].

## Conclusion

As there is no available randomized trial comparing types of stents, or treated and untreated patients [24], strong consideration should be given towards the establishment of at least a national, if not international database of patients undergoing airway stent placement for benign lesions. Analysis of this database may well allow us to not only to establish clinical indications, patient treatment pathways [32] and stent choice and techniques, but also to ultimately maximize treatment efficacy in this unfortunate group of patients.

Nevertheless, in the meantime, SEMAS could be considered as a therapeutic option for inoperable central airway lesions, especially in patients with medical co-morbidities. However, in light of the complication rate observed in this series overall, careful consideration should be given to their use, as treatment with SEMAS may necessitate lifelong follow-up and visual surveillance of the SEMAS as clinically indicated.

## Abbreviations

FDA: Food and Drug Administration; SEMAS: self-expandable metallic stent; FB: flexible bronchoscopy; RB: rigid bronchoscopy; CPAP: continuous positive airway pressure

## Competing interests

The author(s) declares that they have no competing interests.

## Authors' contributions

ALC participated in the study design and data collection, and drafted the manuscript. MMJ participated in the study design, data collection, drafting of the manuscript, and performed the statistical analyses. RPA participated in the draft of the manuscript and interpretation of data. TEA participated in the study design and in drafting the manuscript. All authors read and approved the final manuscript.

## Pre-publication history

The pre-publication history for this paper can be accessed here:


